# Dietary Fiber and the Human Gut Microbiota: Application of Evidence Mapping Methodology

**DOI:** 10.3390/nu9020125

**Published:** 2017-02-10

**Authors:** Caleigh M. Sawicki, Kara A. Livingston, Martin Obin, Susan B. Roberts, Mei Chung, Nicola M. McKeown

**Affiliations:** 1 Nutritional Epidemiology, Jean Mayer USDA Human Nutrition Research Center on Aging at Tufts University, Boston, MA 02111, USA; caleigh.sawicki@tufts.edu (C.M.S.); kara.livingston@tufts.edu (K.A.L.); 2 Friedman School of Nutrition Science and Policy, Tufts University, Boston, MA 02111, USA; 3 Nutrition & Genomics Laboratory, Jean Mayer USDA Human Nutrition Research Center on Aging at Tufts University, Boston, MA 02111, USA; martin.obin@tufts.edu; 4 Energy Metabolism Laboratory, Jean Mayer USDA Human Nutrition Research Center on Aging at Tufts University, Boston, MA 02111, USA; susan.roberts@tufts.edu; 5 Nutrition/Infection Unit, Department of Public Health and Community Medicine, Tufts University School of Medicine, Boston, MA 02111, USA; Mei_Chun.Chung@tufts.edu

**Keywords:** dietary fiber, gut microbiota, evidence map, colonic fermentation, oligosaccharides, resistant starch, cereal fiber, *Bifidobacteria*, *Lactobacilli*

## Abstract

Interest is rapidly growing around the role of the human gut microbiota in facilitating beneficial health effects associated with consumption of dietary fiber. An evidence map of current research activity in this area was created using a newly developed database of dietary fiber intervention studies in humans to identify studies with the following broad outcomes: (1) modulation of colonic microflora; and/or (2) colonic fermentation/short-chain fatty acid concentration. Study design characteristics, fiber exposures, and outcome categories were summarized. A sub-analysis described oligosaccharides and bacterial composition in greater detail. One hundred eighty-eight relevant studies were identified. The fiber categories represented by the most studies were oligosaccharides (20%), resistant starch (16%), and chemically synthesized fibers (15%). Short-chain fatty acid concentration (47%) and bacterial composition (88%) were the most frequently studied outcomes. Whole-diet interventions, measures of bacterial activity, and studies in metabolically at-risk subjects were identified as potential gaps in the evidence. This evidence map efficiently captured the variability in characteristics of expanding research on dietary fiber, gut microbiota, and physiological health benefits, and identified areas that may benefit from further research. We hope that this evidence map will provide a resource for researchers to direct new intervention studies and meta-analyses.

## 1. Introduction

According to the 2009 Codex Alimentarius definition of dietary fiber, which aims to unify the definition among all countries, dietary fiber includes all carbohydrate polymers of three or more monomeric units that resist digestion in the small intestine [1,2]. A further stipulation of this definition is that isolated or chemically synthesized fibers need to show a physiological health benefit. Epidemiological evidence consistently shows that higher intake of dietary fiber is associated with a reduced risk of chronic diseases, such as cardiovascular disease (CVD), type 2 diabetes, and cancer [3]. However, new research is interested in the role of the gut microbiota with respect to observed beneficial effects [4,5].

Research on the human gut microbiota, sometimes referred to as the “forgotten organ” [6], has exponentially increased over the past decade with recent advances in technology. There has been growing evidence that the microbiota not only produces metabolites that can influence host physiology, but these metabolites also play an integral role in the host immune system and metabolism through a complex array of chemical interactions and signaling pathways [7,8,9]. These interactions can greatly impact host health and risk of disease [7,10], and the microbiota have been linked to numerous diseases such as irritable bowel syndrome (IBS), asthma, allergy, metabolic syndrome, diabetes, obesity, cardiovascular disease, and colorectal cancer [11]. 

A number of factors can cause the composition of the microbiota to shift, including changes in diet [10]. Consumption of dietary fiber has been shown to influence the gut microbiota by altering bacterial fermentation, colony size, and species composition [12]. Non-digestible carbohydrates are the primary energy source for most gut microbes and, therefore, can directly impact those species that heavily depend on that substrate [13]. There can also be indirect impacts through cross-feeding, where some types of microbes depend on the by-products, or metabolites, of other types of microbes [14,15]. In addition to substrate availability, the magnitude and diversity of the microbiota are also greatly influenced by other aspects of the gut environment, including pH, host secretions, and transit time [11,12,16,17,18,19]. While certain dietary components play an important role in the gut environment, products of microbial fermentation can also have an influence [10,20]. For example, consumption of fermentable dietary fiber will provide substrates for microbial activity but will also increase the concentrations of fermentation products, such as short-chain fatty acids (SCFAs). A buildup of SCFAs subsequently lowers the colonic pH, which can then have dramatic effects on the composition of the microbiota [16,20,21]. Therefore, the relationship between diet, the gut microbiota, microbial activity, and gut physiology is complex.

The distribution of different strains or species of bacteria within the gut will determine the metabolic profile of the microbiota, which could have potential physiologic effects on health [10]. SCFAs, such as butyrate, acetate, and propionate produced by the fermentation of dietary fibers, may play a role in energy homeostasis, immune function, and host-microbe signaling [7,22,23], and prevention of diseases, such as bowel disease, colon cancer, and metabolic syndrome [20,24,25,26]. Therefore, fiber-induced modulation of the gut microbiota has gained interest for its potential impact on health and disease [27]. However, it is not well understood how and to what extent these changes may happen in a predictable way [28]. The first step toward answering these questions is to gather and summarize the current literature on dietary fiber and the gut microbiota, which can be done effectively using evidence mapping.

Evidence mapping is a new technique being applied in nutritional epidemiology to review and characterize the published research on a broad topic of interest, allowing for the identification of gaps and prioritizing new research questions [29,30,31]. Evidence maps may be considered as the first few steps in a systematic review but are generally more comprehensive in the scope of the research question [31,32]. Instead of a specific, targeted question, an evidence map aims to determine the research “landscape” of the topic area. Evidence mapping can provide a context for systematic reviews and meta-analyses by presenting a wide range of study designs and methods being utilized in the area of interest [29]. While systematic reviews are the method of choice for synthesizing study results, evidence mapping is a more efficient methodology for visualizing the evidence and is a particularly useful technique in fast-paced or rapidly growing areas of research, such as the human gut microbiota.

Our objective was to describe existing research on dietary fiber intake and the gut microbiota. Through the creation of an evidence map, we identify potential gaps in the research and highlight areas where new hypotheses may be addressed in future studies. Furthermore, we extended our evidence mapping to summarize broad study findings in a focused area regarding the effects of the oligosaccharide interventions on the gut microbial composition. In doing so, we demonstrate how this evidence map can be used as a platform to build on the existing evidence by answering the following two questions: (1) Can we identify specific gut microbial species that are modulated by dietary fiber? (2) Is there evidence that modulation of the gut microbiota is correlated with fermentation or physiological effects on host health?

## 2. Materials and Methods

Evidence mapping involves three major steps: (1) clearly defining a topic area and setting criteria around the questions of interest; (2) systematically searching for and selecting relevant studies based on pre-defined criteria, such as study design and outcomes of interest and, thereby, creating a “map” of available evidence; and (3) reporting on study characteristics and the extent of existing research [30,31,32].

To develop the dietary fiber and human gut microbiota evidence map, we utilized a newly developed Dietary Fiber Database (Version 3.0), containing data on published dietary fiber interventions [33]. The database, housed in the Systematic Review Data Repository (SRDR) [34], contains descriptive data extracted from dietary fiber intervention studies that were identified by a systematic literature search. It includes all human studies published from 1946 to May 2016 that examined the effect of dietary fiber on at least one of nine pre-defined physiological health outcomes. For the complete list of the inclusion/exclusion criteria, including the nine health outcomes, refer to Supplementary Materials Table S1.

The database includes two specific outcomes related to the gut microbiota: (1) modulation of colonic microflora; and (2) colonic fermentation/short-chain fatty acid concentration. Keywords used to identify these specific outcomes in the development of the database are provided in the Supplementary Methods. Our evidence map is comprised of all publications in the database reporting on at least one of these two outcomes.

Descriptive analyses were performed to examine the range of study designs, fiber interventions, and types of outcomes examined. Because evidence mapping is meant to capture the wider landscape of evidence and is, therefore, more inclusive and less homogenous than is usually required for a meta-analysis, results are specifically not represented. Due to the large variety of fiber interventions identified, fiber intervention exposures were grouped into categories according to structure or source, depending on how they were described in the original publication. If the same fiber intervention was given at different doses within the same study, that fiber type was only counted once for that study. 

Microbiota outcomes identified by the database were examined in more detail and were re-classified into three categories: (1) fermentation, which included measures of SCFAs, breath markers (such as H_2_ and CH_4_), bacterial enzyme activity and metabolites, bile acid metabolism, and fiber digestibility (measured by fecal recovery); (2) bacterial composition, which included relative or absolute bacterial counts; and (3) colonic and fecal pH.

Weighted scatter plots were used to visualize the available evidence on different fiber types by outcome groups and sample size. Each bubble in the plot represents a single publication with the size of the bubble corresponding to the study sample size. Publications may be represented more than once throughout the plot if multiple fiber interventions or outcomes were reported but are not repeated within any single cross-sectional area. 

To further explore the information captured in this evidence map, we isolated publications on the top most reported fiber type, oligosaccharides. We examined oligosaccharides in relation to bacterial composition and extracted more detailed information on the study characteristics, bacterial strains and/or species identified in the publication and the direction of change in strain/species frequency (increased, decreased, or remained the same) in response to the fiber intervention. 

## 3. Results

A total of 188 distinct studies with at least one outcome related to the gut microbiota were identified in the Dietary Fiber Database (Version 3.0). The study design and population characteristics of these studies are summarized in Table 1.

The majority (96%) were randomized, controlled studies, with only two studies not randomized, and three studies with unknown randomization. The majority of studies used a crossover design compared to a parallel design (67% randomized, crossover; 29% randomized, parallel; and 1% combination of crossover and parallel designs). The size of the study samples ranged from 4 to 435 subjects, but the majority of studies (87%) had fewer than 50 subjects. Few studies (14%) had an intervention duration exceeding four weeks. Most (67%) lasted 1–4 weeks, and 19% were acute feeding interventions, which usually consisted of a single test meal. The subjects were described as healthy in the majority of studies (81%). Fewer studies involved subjects that were overweight or obese (4%), diabetic (1%), hyperlipidemic (3%), had metabolic syndrome or “at-risk” for metabolic syndrome (4%), digestive issues (3%), or risk factors for developing colon cancer (2%).

Within the 188 studies, 47 different fiber types were captured. These fiber interventions fell into 11 different categories, as detailed in Table 2.

The fibers most frequently studied were oligosaccharides (20% of studies), resistant starch (16%), and chemically synthesized fibers (15%), followed closely by inulin (13%), bran (13%) and cereal fiber (11%). The study design characteristics for studies examining the top three fibers are also presented in Table 1. Notably, resistant starch had a higher proportion of studies with a sample size of fewer than 10 subjects, while oligosaccharides were more often examined in studies of much larger sample sizes and longer duration. 

Table 3 reports the frequency and percentage of three major microbiota outcomes: bacterial composition (47% of studies), colonic/fecal pH (32%), and fermentation (76%). Fermentation is further broken down by the specific measurement used to determine the degree of fermentation. SCFA concentration (52%) and breath gas excretion (27%) were the most commonly measured markers of fermentation, but others included bacterial enzyme activity, bile acid metabolism, and fecal starch recovery.

**Figure 1:**
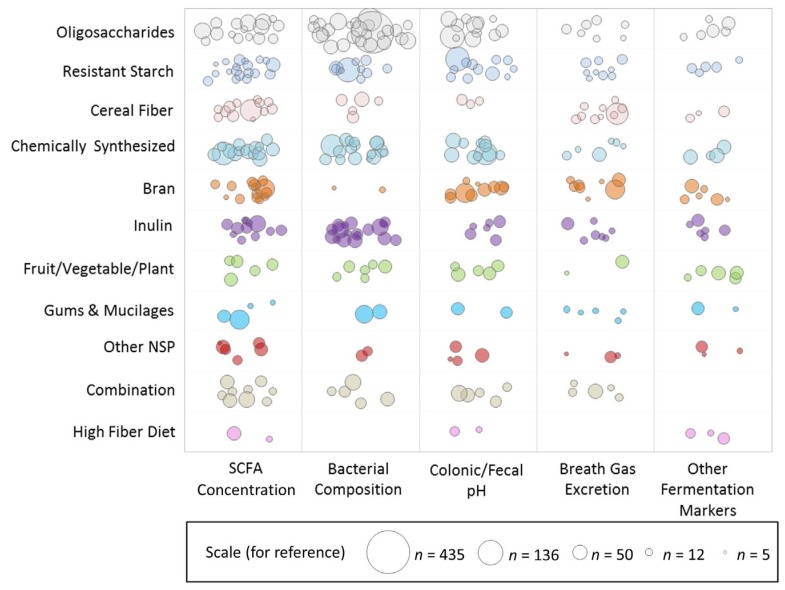
Weighted scatter plot of microbiota outcomes by fiber group. Each bubble in the plot represents a single publication with the size of the bubble corresponding to the study sample size. Studies may be represented more than once throughout the plot if multiple fiber interventions or outcomes were reported but are not repeated within any single cross-sectional area. Note that the outcome effect is not represented in this graphic, i.e., this does not reflect the effect of the fiber on the outcome.

Figure 1 is a weighted scatter plot of the microbiota outcomes by fiber group. It provides a visual representation summarizing the research activity in this field. For example, while SCFA concentration and bacterial composition are studied often, fewer and less sizeable studies measure breath gas excretion and other markers of fermentation. Specific gaps in the research are readily identified. Notably, we can see that there are currently no published studies examining the effect of a high fiber whole-diet intervention on bacterial composition of the microbiota. This plot also shows active areas of interest. We can see, for example, that a large number of relatively larger studies have been published on bacterial composition and oligosaccharide interventions. 

**Figure 2:**
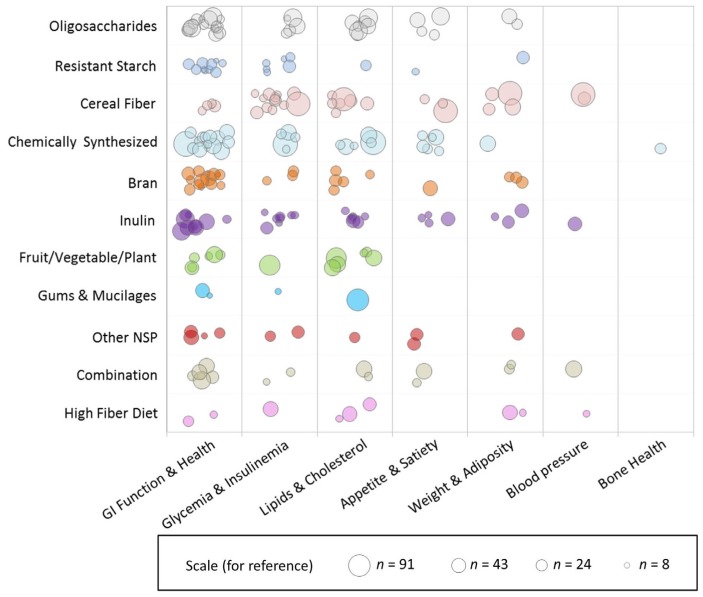
Weighted scatter plot of other physiological health outcomes by fiber group. Each bubble in the plot represents a single publication with the size of the bubble corresponding to the study sample size. Studies may be represented more than once throughout the plot if multiple fiber interventions or outcomes were reported but are not repeated within any single cross-sectional area. Note that the outcome effect is not represented in this graphic, i.e., this does not reflect the effect of the fiber on the outcome.

Figure 2 is a weighted scatter plot displaying the other physiological health outcomes captured in this evidence map. Not surprisingly, gastrointestinal (GI) health, which includes measures of fecal bulking, laxation, and transit time, is very frequently studied along with the gut microbiota, but there is less evidence on satiety, adiposity, and blood pressure. There is also just one study published so far examining bone health in the context of dietary fiber and the gut microbiota, a very new emerging area of interest. Supplementary Materials Figures S1 and S2 present weighted scatter plots similar to those in Figure 2, restricted by study duration. The acute studies (Figure S1) exclusively examine the short-term fermentation response by measuring SCFA concentration and/or breath gas excretion, most frequently in cereal fibers, whereas studies of greater duration (Figure S2) examine more outcomes in a larger array of fiber types. Notably, however, there are no longer duration (>4 weeks) studies on resistant starch.

We extended our evidence mapping of the fiber–microbiota research landscape by examining one of the most active areas in more detail: oligosaccharide interventions and bacterial composition. There were 26 studies (from 25 publications) on this topic. Three of the studies utilized a dose of antibiotics to specifically examine the use of oligosaccharides to assist recolonization of the gut, and, for comparison purposes, we excluded these from this sub-analysis. Details of the remaining 23 studies are shown in Table 4. Studies were published between 1996 and 2015, and all were randomized controlled trials (9 parallel, 14 crossovers). Intervention durations ranged from 1 to 12 weeks, and sample size ranged from 15 to 136 (mean of 46) subjects. Notably, only one study recruited subjects that were overweight with metabolic syndrome, and one study recruited subjects that were overweight, while all the other studies reported on healthy subjects. Most studies had a similar age range, but there was one study in children and one specifically in older adults. 

Among these 23 studies, there are 26 fiber interventions: eight fructooligosaccharides (FOS), nine galactooligosaccharides (GOS), six arabinoxylan-oligosaccharides (AX-OS), two xylo-oligosaccharide (XOS), and one soybean oligosaccharide. All but one study found a bifidogenic effect of oligosaccharides, with doses as small as 1.4 g/day (XOS) and a range of treatment forms, including tablets, beverages, and whole foods. Many studies also examined oligosaccharide impacts on *Lactobacilli* or *Lactobacillus–Enterococcus* frequency, with 19 studies reporting no effect, three reporting a positive effect, and one reporting a negative effect. Few publications reported decreases in bacterial strains/species, but among those that did, it was most often *Bacteroides*, with four studies finding a significant decrease in response to a GOS intervention. However, two intervention studies found no effect of GOS on *Bacteroides*, five interventions using other oligosaccharides also found no effect, and one XOS intervention found a significant increase in *Baceroides fragilis*. There were a number of other strains/species reported; however, it is important to note that some studies used targeted culturing techniques, whereas others used DNA sequencing to attempt to identify all species present. 

Table 5 summarizes the other physiological outcomes examined within these studies. Ten out of the 26 interventions also significantly increased markers of fermentation, but only one intervention had a significant effect on fecal bulking, and only two had significant effects on transit time. Of the other physiologic outcomes examined in these studies, most were related to GI health. Only a few studies measured lipids, glucose, or insulin, and of those that did, only one study found a significant effect on total cholesterol and insulin. 

## 4. Discussion

Observational/epidemiologic evidence shows that diets higher in fiber are associated with reduced risk of certain chronic diseases, such as heart disease, diabetes, and obesity [3,57], and these may be related to the effect of dietary fiber on the gut microbiota [4,5]. However, the present evidence map reveals that there is insufficient data from well-controlled dietary fiber interventions that study the gut microbiota in relation to intermediate risk factors of cardiometabolic disease or in relation to chronic conditions such as obesity. In fact, we found little evidence on the intersection of dietary fiber, the microbiota, and adiposity. Much of the current literature has shown positive effects of dietary fiber on gut function or beneficial bacterial species, or positive effects of dietary fiber on specific health outcomes, but few seem to be directly measuring these outcomes together, to provide evidence of a dietary fiber-modulated gut microbiota and health outcome [58,59].

Over the last 25 years, there has been a rapid increase in interest on dietary fiber and the microbiota, particularly with respect to prebiotics, such as oligosaccharides and inulin, as well as chemically synthesized fibers such as Polydextrose (PDX), soluble corn fiber, and PolyGlycopleX (PGX). From this map, we can see that the most actively researched fibers are oligosaccharides, resistant starch, and chemically synthesized fibers, followed closely by inulin, bran, and cereal fiber (Table 2), and the most common measures of the gut microbiota are SCFA concentration and bacterial composition (Table 3). The fiber and outcome most frequently studied together were oligosaccharides and bacterial composition, and we, therefore, examined these studies in more detail in summarizing our evidence map. 

Oligosaccharides are short-chain saccharide polymers, generally made up of 3–10 carbohydrate monomers [60,61], and are known for their prebiotic activity. Prebiotics are defined as non-digestible foods that, when metabolized, alter the composition and/or activity of the microbiota in a such a way that promotes the health of the host [4,62,63]. Randomized controlled trials have consistently shown that oligosaccharides, and FOS in particular, increase *Bifidobacterium* (Table 4), a genus of oligosaccharide fermenting gut bacteria that may be beneficial to human health [64,65,66]. Despite the considerable number of studies showing this bifidogenic effect, few have actually examined direct relationships of this modulation of the gut microbiota to other physiological health outcomes. In our sub-analysis on oligosaccharides, we found that only five [43,45,51,52,53] of the 26 oligosaccharide interventions measuring bacterial composition also measured changes in lipids, and only two [51,52] measured glucose and insulin response. Additionally, only one study [52] found a small statistically significant beneficial effect on total cholesterol and insulin, while the rest found no beneficial effect. These findings are consistent with reviews by McRorie et al. [67,68,69,70], which conclude that clinical evidence does not support a link between soluble, non-viscous, readily fermentable fibers (such as oligosaccharides) and physiological health benefits on cholesterol, glycemic control, or laxation. Rather, these benefits are attributed to the physical properties of soluble, viscous/gel-forming fibers that are not readily fermented (such as beta glucan and raw guar gum). 

However, readily-fermented fiber types, such as oligosaccharides and resistant starch, may have other important physiologic effects via the metabolites produced from microbial fermentation. The most studied products of fermentation are SCFAs, mainly butyrate, propionate, and acetate [25]. Up to 95% of SCFAs are absorbed by the colonocytes of the large intestine [20], and recent evidence has shown that they may play a role in health and prevention of disease, such as bowel disease, colon cancer, and metabolic syndrome [20,24,25,26]. SCFAs have been shown to affect gut health, immune function, energy metabolism, stimulation of the sympathetic nervous system, and serotonin release [4,7,20]. 

This evidence map also highlights areas where evidence is lacking. For instance, high-fiber diet interventions, where total dietary fiber was increased from a variety of sources, have the least number of publications. As more emphasis is put on the importance of dietary patterns rather than individual foods or food components [71], more whole-diet intervention studies need to be conducted to understand how these relationships among diet, the gut microbiota, and health work in the context of a whole diet. With respect to microbiota outcomes, fewer studies have measured bacterial enzyme activity, which may be more important than simply measuring changes in the bacterial composition because some strains or species may alter their function, and, therefore, their metabolites, in response to changes in the gut environment rather than their absolute number. Well-controlled human intervention trials incorporating “next generation” metagenomics, meta-transcriptomics and metabolomics will be vital to further understanding these changes in microbial activity. In addition, the microbiota composition or activity may be altered in people with chronic diseases such as obesity [58], and future research should consider whether diet may have differential effects depending on underlying health status. Based on this evidence map, the majority of fiber research on the gut microbiota was conducted in healthy adults (81%).

The definition of fiber has been a moving target, and fibers, being one of the most heterogeneous groups of associated molecules, have been categorized in many different contexts, including by source, structure, or physical properties (solubility, fermentability, etc.). Further, fibers may be delivered/consumed as isolated supplements, but they are more often consumed intact in whole foods (such as in raw fruits and vegetables), or in processed foods (including processes such as cooking, milling, and baking). The food matrix is important to consider because other components of the food, such as phytochemicals, may provide synergistic effects [72,73], and the degree of processing can alter the structure and physical attributes of the fiber [70]. All of these factors contribute to the type and extent of microbial utilization [28]. Therefore, it is important for future studies to describe the dietary fiber intervention in as much detail as possible, and, where applicable, define the characteristics of the fibers being studied.

Creating an updatable evidence map of microbiota-related outcomes allows researchers to obtain a more global view of the research landscape, including its history, current trajectory, and specific areas or research questions lacking data or consensus. Since the Dietary Fiber Database used for this evidence map captures literature going back to 1946, it is important to note that the literature in the database often represents evolving knowledge about particular fiber types. This is both a strength and limitation of the database and the evidence mapping process overall, and it highlights the importance of reviewing the totality of available evidence. For instance, bran is well represented in the evidence map, and although the effect of bran on the microbiota was of interest in earlier publications, it is now well-established that insoluble bran is not very readily fermented and may have less of an effect on the microbiota. Instead, interest has shifted toward more fermentable fibers such as the prebiotics and chemically synthesized fibers. 

Unlike systematic reviews, which generally address a narrower, focused question with extensive quality analysis and risk of bias assessment, the primary goal of evidence mapping is to identify patterns and provide a broader context within which systematic reviews may occur. As such, in Table 4 and Table 5, we did not provide information on effect size of the significance, as this information was not reported in the published fiber database [33]. Furthermore, because of the complex nature of dietary fiber and the size of the literature on dietary fiber, the database which this evidence map is based on has some inherent limitations, which are detailed in a separate publication [33]. Notably, the database was limited to publications in PubMed, and studies were only included if there was a well-defined dietary fiber intervention with a concurrent control. Further, because this database was designed to specifically capture certain health outcomes, other publications of interest to microbiota research may not be represented. 

A major strength of this evidence map is that it is a cost effective way of summarizing the data on dietary fiber and the gut microbiota. For instance, the data presented in Table 4 and Table 5 may be used to guide future work, such as a meta-analysis, which would provide more specific information to quantify statistical significance. We were able to use a previously created database in order to efficiently identify potentially relevant literature. Although we closely reviewed the subset of relevant literature identified via the database, we saved significant resources in conducting initial, broad searches which would inevitably yield a large amount of irrelevant literature to screen through. The result is a useful platform to visualize the current evidence, which can be used to summarize the volume of existing research, generate new hypotheses, direct systematic reviews and meta-analyses, and can be continually updated.

## 5. Conclusions

In conclusion, this evidence map summarizes the existing literature on dietary fiber interventions and the human gut microbiota. This is a rapidly growing area of interest, but well-controlled human interventions are needed to support the associations being seen in animal and observational studies. We hope that this evidence map will provide a resource for researchers to direct new intervention studies and meta-analyses. 

## Figures and Tables

**Table 1:** Study Design Characteristics.

Characteristic, *n* (% of Studies)	Total	Top Three Fiber Types		
		Oligosaccharides	Resistant Starch	Chemically Synthesized
***n***	188	38	30	28
**Design**				
Randomized, parallel	54 (29%)	14 (37%)	3 (10%)	10 (36%)
Randomized, crossover	127 (67%)	24 (63%)	27 (3%)	16 (57%)
Randomized, combined parallel and crossover	2 (1%)	0 (0%)	0 (0%)	1 (4%)
Non-Randomized	2 (1%)	0 (0%)	0 (0%)	0 (0%)
Unspecified Randomization	3 (2%)	0 (0%)	0 (0%)	1 (4%)
**Sample size**				
Less than 10	19 (10%)	1 (3%)	6 (20%)	2 (7%)
10 to 49	145 (77%)	29 (76%)	23 (77%)	23 (82%)
50 to 100	20 (11%)	6 (16%)	0 (0%)	2 (7%)
More than 100	4 (2%)	2 (5%)	1 (3%)	1 (4%)
**Duration**				
Acute (<1 week)	36 (19%)	4 (11%)	9 (30%)	6 (21%)
1–4 weeks	126 (67%)	26 (68%)	21 (70%)	20 (71%)
1–6 months	25 (13%)	8 (21%)	0 (0%)	2 (7%)
More than 6 months	1 (1%)	0 (0%)	0 (0%)	0 (0%)
**Diet type**				
Acute	36 (19%)	4 (11%)	9 (30%)	6 (21%)
Isocaloric/Maintenance	115 (61%)	26 (68%)	17 (57%)	12 (43%)
Weight Loss	2 (1%)	0 (0%)	0 (0%)	0 (0%)
Other/Unspecified	35 (19%)	8 (21%)	4 (13%)	10 (36%)
**Age**				
Adults (≥17 years *)	185 (98%)	37 (97%)	30 (100%)	27 (96%)
Adolescents (12–17 years)	1 (1%)	0 (0%)	0 (0%)	1 (4%)
Children (3–11 years)	2 (1%)	1 (3%)	0 (0%)	0 (0%)
**Baseline Health**				
Healthy	153 (81%)	34 (89%)	26 (87%)	27 (96%)
Overweight or Obese	7 (4%)	1 (3%)	0 (0%)	1 (4%)
Diabetic	1 (1%)	0 (0%)	0 (0%)	0 (0%)
Metabolically at Risk	8 (4%)	1 (3%)	2 (7%)	0 (0%)
Hyperlipidemia	6 (3%)	1 (3%)	1 (3%)	0 (0%)
GI/Digestive Issues	6 (3%)	0 (0%)	0 (0%)	0 (0%)
Other	7 (4%)	1 (1%)	1 (3%)	0 (0%)
**Region**				
Asia	6 (3%)	2 (5%)	1 (3%)	3 (11%)
Australia/New Zealand	16 (8%)	1 (3%)	7 (23%)	1 (4%)
Europe	114 (61%)	31 (81%)	17 (57%)	12 (43%)
North America	51 (27%)	4 (11%)	5 (17%)	12 (43%)
South America	1 (1%)	0 (0%)	0 (0%)	0 (0%)

* Only one study had an age range of 17–61 years, all other studies in adults included subjects ≥18 years.

**Table 2:** Fiber types (total studies = 188), 324 unique exposures.

Group	Studies (%)	Fiber Types	*n*
Oligosaccharide	38 (20%)	Fructooligosaccharide	22
		Galactooligosaccharide	11
		Arabinoxylan-oligosaccharides	6
		Xylo-oligosaccharide	2
		Soybean oligosaccharides	1
Resistant Starch	30 (16%)	Resistant starch type 1	1
		Resistant starch type 2 ^a^	20
		Resistant starch type 3	11
		Resistant Starch, mixed or unspecified	4
Chemically synthesized	28 (15%)	Polydextrose	12
		Dextrin ^g^	9
		Soluble corn fiber	7
		PolyGlycopleX (PGX)	2
		Resistant starch type 4	2
		Microcrystalline cellulose	1
		Solubilized potato polysaccharide	1
		Pullulan	1
		Butyrylated high amylose maize starch	1
Inulin	25 (13%)	Inulin	18
		Oligofructose-enriched inulin (OF-IN)	7
Bran	24 (13%)	Wheat Bran	12
		Oat Bran	9
		Corn bran	2
		Barley bran	1
		Rye Bran	1
		Bran	2
Cereal fiber	21 (11%)	Cereal fiber, wheat ^b^	9
		Cereal fiber, barley ^c^	8
		Cereal fiber, oat ^d^	4
		Cereal fiber, rye ^e^	4
		Cereal fiber ^f^	3
Fruit/Vegetable/Plant fibers	15 (8%)	Vegetable fiber	6
		Lupin Kernel Fiber	3
		Sugar cane fiber	2
		Sugar Beet fiber	1
		Bean fiber	1
		Citrus fiber	2
		Fruit fiber	1
Combination	13 (7%)	Combination/Mixture	13
Gums and Mucilages	10 (5%)	Gums ^h^	7
		Psyllium ^i^	6
Other non-starch polysaccharides	9 (5%)	Pectin	4
		Cellulose	3
		Hemicellulose ^j^	3
		Beta-glucan, barley	1
		Polysaccharide, non-starch	1
High fiber diet	4 (2%)	Dietary fiber	4

More specific fiber types described include ^a^ high-amylose maize starch; ^b^ whole-grain wheat; ^c^ barley flour, barley kernels; ^d^ oat kernels; ^e^ whole-grain rye, rye kernels; ^f^ whole-grain, mixture, or unspecified cereal fiber; ^g^ wheat dextrin, resistant dextrin, resistant maltodextrin, soluble fiber dextrin; ^h^ guar gum, gum arabic; ^i^ ispaghula, Metamucil; ^j^ arabinogalactan, xylans, glucomannan.

**Table 3:** Microbiota outcomes (total studies = 188).

Outcome Group	Studies (%)
Fermentation	142 (76%)
SCFA concentration	98 (52%)
Breath gas excretion	50 (27%)
Bacterial enzyme activity	18 (10%)
Bile acids	15 (8%)
Fecal fiber/starch recovery	13 (7%)
Bacterial composition	88 (47%)
Colonic/fecal pH	60 (32%)

**Table 4:** Characteristics of oligosaccharide interventions and direction of evidence on bacteria composition.

Reference	*n*	Design	Duration	Age, Mean (Range)	% Male	BL Health	BMI, Mean (Range)	Fiber Type (g/Day)	Form	Control	Method	Bacterial Composition Reported Measures
[35]	20	RCT, P, DB	12 days	(22–39)	50	Healthy	NR	FOS (12.5)	NR (3 oral doses)	Saccharose placebo	Stool (whole), Wilkins-Chalgren agar, Beerens’ medium	↑ *Bifidobacteria*
												NS Total anaerobes
[36]	40	RCT, P	7 days	29.6 (18–47)	45	Healthy	NR	FOS (2.5, 5, 10, 20)	Powder	Saccharose powder	Stool (whole), Wilkins-Chalgren, Beeren’s medium	↑ *Bifidobacteria* (at doses 5–20 g)
												NS Total anaerobes
[37]	15	RCT, C, DB	3 weeks	NR	~47	Healthy	NR	FOS (2.5)	Biscuits	Matched biscuits without FOS	Stool (partial), PCR-DGGE (denaturing gradient gel electrophoresis), fluorescent in situ hybridization (FISH)	NS *Bifidobacteria*
												NS Lactobaccilli
												NS Lactose-fermenting enterobacteria
												NS Total enterobacteria
												NS Enterococci
								GOS (2.5)	Biscuits			NS *Bifidobacteria*
												NS *Lactobaccilli*
												NS Lactose-fermenting enterobacteria
												NS Total enterobacteria
												NS *Enterococci*
[38]	136	RCT, P, DB	7 days	~30 (~18–54)	~41	Healthy	NR	FOS (2.5, 5.0, 7.5, 10)	NR (2 oral doses)	Sucrose and fully digestible maltodextrin placebo	Stool (partial), Wilkins-Chalgren agar. Beerens’s medium, Bacteroides Bile Esculin agar, Lactobacillus agar, MRS agar, and McConkey agar	↑ *Bifidobacteria*
												NS Total anaerobes
												NS *Lactobacillus*
												NS *Baceroides*
												NS *Enterobacteria*
								GOS (2.5, 5.0, 7.5, 10)	NR (2 oral doses)			↑ *Bifidobacteria*
												NS Total anaerobes
												NS *Lactobacillus*
												NS *Baceroides*
												NS *Enterobacteria*
								SB-OS (2.5, 5.0, 7.5, 10)	NR (2 oral doses)			↑ *Bifidobacteria*
												NS Total anaerobes
												NS *Lactobacillus*
												NS *Baceroides*
												NS *Enterobacteria*
[39]	39	RCT, P, DB	30 days	60.4	0	Healthy	NR	FOS (7)	Cereal bars and gelified milk	Matched cereal bars and gelified milk without FOS	Stool (partial), temperature-gradient gel electrophoresis (TTGE), FISH	↑ *Bifidobacterium* spp.
												↑ Bifidobacterium Animalis and related species
												NS Bacteroides and relatives
												NS Clostridium coccoides-Eubacterium rectale cluster
												NS Faecalibacterium prausnitzi subgroup
												NS Lactobacillus-Enterocococcus group
												NS Atopobium group
[40]	34	RCT, C, DB	2 weeks	27.7	100	Healthy	23.2	FOS (20)	Beverage (lemonade)	Matched lemonade with sucrose placebo	Stool (whole), RT-qPCR	↑ *Bifidobacteria*
												↑ *Lactobacilli*
												NS *E. coli*
[41]	40	RCT, P	7 days	29	~45	Healthy	NR	FOS (2.5, 5.0, 7.5, 10)	Tablet	Sucrose and fully digestible maltodextrin placebo	Stool (partial), Wilkins-Chalgren agar, Beerens‘ medium, MRS agar, BBE agar, McConkey agar	↑ *Bifidobacteria* (all doses)
												↑ Total Anaerobes (10 g only)
												NS *Lactobacilli*
												NS *Bacteroides*
												NS *Enterobacteria*
[42]	30	RCT, C, DB	7 days	36.3 (21–59)	~40	Healthy	NR	GOS (3.6, 7)	Powder, mixed with water	Matched sucrose placebo powder	Stool (whole), FISH	↑ *Bifidobacterium*
												↑ Clostridium perfringens- histolyticum subgroup (3.6 g only)
												NS *Lactobacillus-Enterococcus* spp.
												NS Bacteroides-prevotella
	29			32.5 (19–55)	~45	Healthy	NR	GOS (7)	Powder, mixed with water	Matched powder without GOS		↑ *Bifidobacterium*
												↓ Bacteroides-prevotella
												NS *Lactobacillus-Enterococcus* spp.
												NS Clostridium perfringens-histolyticum subgroup
[43]	44	RCT, C, DB	10 weeks	69.3 (64–79)	~36	Healthy	(22–31)	GOS (5.5)	Powder, mixed with water	Matched maltodextrin placebo	Stool (partial), FISH	↑ Bifidobacterium spp.
												↑ *Lactobacillus-Enterococcus* spp.
												↑ Clostridium coccoides-Eubacterium rectale group
												↓ *Bacteroides* spp.
												↓ Clostridium histolyticum group
												↓ *Escherchia coli*
												↓ *Desulfovibrio* spp.
[44]	64	RCT, P, DB	30 days	33 (22–51)	~41	Healthy	NR	FOS (5)	Powder, used to prepare a jelly	Commercial dessert (jelly, lemon flavored)	Stool (partial), Beerens’ agar, Chromocult Coliform agar, Slanetz and Bartley medium, Rogosa agar, Wilkins-Chalgren anaerobe agar with 5% (v/v) defibrinated horse blood and G-N anaerobe selective supplement (OXOID), Perfringens agar with d-cycloserine.	↑ *Bifidobacterium* spp.
												↓ Total coliforms
												↓ *Escherichia coli*
												NS Total aerobes
												NS *Enterococcus* spp.
												NS Total anaerobes
												NS *Bacteroides* spp.
												NS *Lactobacillus* spp.
												NS *Clostridium perfringens*
[45]	20	RCT, C	3 weeks	24	30	Healthy	20.9	AX-OS (10)	Beverage (orange juice drink)	Matched maltodextrin placebo beverage	Stool (partial), real-time PCR, real-time PCR TaqMan, real-time PCR SYBR Green technology	↑ *Bifidobacteria*
												↑ *Bifidobacterium adolescentis*
												↓ *Lactobacilli*
												NS Total bacteria
												NS Roseburia-Eubacterium rectale
												NS *Enterobacteria*
[46]	39	RCT, C, DB	3 weeks	58.9 (50–81)	~46	Healthy	26.1 (19.7–38.4)	GOS (~8)	Beverage (orange juice drink)	Matched placebo beverage	Stool (partial), quantitative PCR, FISH	↑ *Bifidobacterium*
												↓ *Bacteroides*
												NS *Total bacteria*
												NS *Lactobacillus*
												NS *Escherichia coli*
												NS Eubacterium rectales group
												NS Clostridium histolyticum group
[47]	60	RCT, P, DB	4 weeks	~20 (18–24)	~43	Healthy	~21.3	X-OS (5)	Beverage (orange juice drink)	Matched wheat maltodextrin placebo beverage	Stool (partial), quantitative PCR	↑ *Bifidobacterium*
												NS *Lactobacillus*
												NS *Peptostreptococcus*
												NS *Clostridium*
												NS *Firmicutes*
												NS *Bacteroidetes*
												NS Faecalibacterium prausnitzii
												NS *Roseburia* spp.
[48]	27	RCT, C, DB	3 weeks	25	~37	Healthy	20.9	AX-OS (2.14)	Wheat/rye bread	Matched wheat/rye or refined wheat bread, no AX-OS	Stool (partial), FISH	↑ *Bifidobacterium*
												NS Total bacteria
												NS Lactobacillus
												NS Lactobacillus rods
												NS Enterobacteriaceae
												NS *Clostridium* histolyticym/lituburiense
[49]	63	RCT, C, DB	3 weeks	42	~52	Healthy	23.3	AX-OS (2.4, 8)	Beverage (non-carbonated soft drink)	Placebo beverage with 0.25 g tricalcium phosphate, no AX-OS	Stool (partial), FISH, 4′-6-diamidino-2-phenylindole (DAPI)	↑ Bifidobacterium (8 g only)
												NS Total bacteria
												NS Lactobacilli
												NS Faecalibacterium prausnitzii
												NS Clostridium histolyticum-lituseburense
												NS Roseburia-Eubacterium rectale
[50]	40	RCT, C, DB	21 days	31.4 (18–55)	50	Healthy	23.3 (18.5–30.0)	AX-OS (2.2)	Wheat/rye bread	Matched wheat/rye bread without AX-OS	Stool (whole), FISH	↑ *Bifidobacterium* spp.
												↑ *Escherichia coli*
												↑ *Lactobacillus-Enterococcus*
												↑ Total bacteria
												↑ Bacteroides
												NS Clostridium histolyticum group
												NS Atopobium-Coriobacterium group
												NS Eubacterium rectale group
												NS Roseburia-Eubacteria
												NS Faecalibacterium prausnitzii cluster
[51]	65	RCT, C, DB	21 days	53.1 (18–75)	46	Healthy	27.8 (18.5–35.0)	AX-OS (2.2, 4.8)	Wheat-based ready-to-eat cereal	Wheat-based ready-to-eat cereal without AXOS	Stool (partial), FISH	↑ Bifidobacterium (4.8 g only, significant dose trend)
												NS Total bacteria
												NS *Lactobacillus* spp.
												NS Bacertoides
												NS Clostridium coccoides
												NS Roseburia intestinalis- Eubacterium rectale group
												NS Faecalibacterium prausnitzii
												NS Clostridium clusters I and II
[52]	48	RCT, C, DB	12 weeks	~44.6	36	OW, metabolic syndrome	~31.4	GOS (5.5)	Powder, mixed with water	Maltodextrin placebo	Stool (partial), FISH	↑ *Bifidobacteria*
												↓ *Bacteroides* spp.
												↓ *Clostridium histolyticum* group
												↓ *Desulfovibrio* spp.
												NS Total bacteria
												NS *Lactobacillus-Enterococcus* spp.
												NS Clostridium coccoides-Eubacterium rectale group
												NS Atopobium cluster
												NS Eubacterium cylindroides
												NS Eubacterium hallii
												NS Beta-proteobacteria
												NS Clostridium cluster IX
												NS Faecalibacterium prausnizii cluster
[53]	28	RCT, C, DB	3 weeks	9.8 (8–12)	64	Healthy	NR	AX-OS (5.0)	Beverage	Placebo beverage with 0.25 g tricalcium phosphate, no AX-OS	Stool (partial), FISH	↑ Bifidobacteria
												NS Clostridium histolyticum/lituseburense
												NS Faecalibacterium prausnitzii
												NS Lactobacillus/Enterococcus
												NS Roseburia/Eubacterium rectale
												NS Total bacteria
[54]	32	RCT, P, DB	8 weeks	~32.4 (21–49)	~34.4	Healthy	~24.6	XOS (1.4, 2.8)	Tablet	Maltodextrin placebo	Stool (partial), 16 rRNA gene sequencing, pyrosequencing	↑ Bifidobacterium
												↑ Total anaerobic flora
												↑ Bacteroides fragilis (2.8 g only)
												↑ Faecalibacterium (2.8 g only)
												↑ Akkermansia (2.8 g only)
												↓ Enterobacteriaceae (placebo only)
												NS Lactobacillus
												NS Clostridium
												NS Clustering
[55]	44	RCT, P, DB	14 d	~37 (18–60)	50	OW	~26.5 (25–28)	GOS (12.0)	Beverage (oolong tea)	Matched beverage with glucose	Stool, real-time quantitative PCR	↑ Bifidobacteria
												NS Total bacteria
[56]	40	RCT, C, DB	10 weeks	70 (65–80)	38	Healthy	NR	GOS (5.5)	Powder, mixed with water	Maltodextrin placebo	Stool (partial), FISH	↑ *Bifidobacterium* spp.
												↑ *Bacteroides* spp.
												NS Atopobium cluster
												NS Clostridium coccoides/*E. rectale*
												NS Clostridium histolyticum group
												NS *Desulfovibrio* spp.
												NS *Escherichia coli*
												NS *Lactobacillus*/*Enterococcus* spp.
												NS Faecalibacterium prausnitzii
												NS Roseburia/Eubacterium rectale
												NS Total bacteria

Abbreviations: AX-OS, arabinoxylan-oligosaccharides; BL, baseline; C, crossover; DB, double-blind; FOS, fructooligosaccharides; GOS, galactooligosaccharides; MS, metabolic syndrome; NR, not reported; NS, no significant change; OW, overweight; P, parallel; RCT, randomized controlled trial; SB-OS, soybean oligosaccharides; X-OS, xylo-oligosaccharides; ~ denotes a value that was calculated or estimated from the data available in the publication; ↑ significantly increased; ↓ significantly decreased.

**Table 5:** Other outcomes reported in oligosaccharide interventions reporting on bacterial composition.

Reference	Fiber Type	Evidence of Fermentation	Evidence of Fecal Bulking	Evidence of Changes in Transit Time	Evidence of Other Changes in Host Physiology
[35]	FOS	S	NS	--	S: GI symptoms (mild bloating)
					NS: Fecal pH
[36]	FOS	NS	--	--	S: GI symptoms (excess flatus)
					NS: Fecal pH
[37]	FOS	S	--	--	NS: GI symptoms
	GOS	S	--	--	NS: GI symptoms
[38]	FOS	--	--	NS	NS: GI symptoms, fecal pH
	GOS	--	--	NS	NS: GI symptoms, fecal pH
	SB-OS	--	--	NS	NS: GI symptoms, fecal pH
[39]	FOS	--	--	--	--
[40]	FOS	S	S	--	S: GI symptoms (flatulence and intestinal bloating)
					NS: fecal water pH
[41]	FOS	--	--	--	S: GI symptoms
					NS: fecal pH
[42]	GOS	--	--	--	--
	GOS	--	--	--	--
[43]	GOS	--	--	--	NS: Total and HDL cholesterol^#^
[44]	FOS	--	--	--	S: GI symptoms
[45]	AX-OS	--	NS	--	NS: Total, LDL, and HDL cholesterol ^#^
[46]	GOS	--	--	--	NS: GI symptoms, stool consistency ^#^
[47]	X-OS	S	--	--	NS: Stool consistency ^#^
[48]	AX-OS	S	--	S	NS: Stool consistency ^#^
[49]	AX-OS	S	--	NS	NS: Total energy intake, total and LDL cholesterol ^#^, stool consistency
[50]	AX-OS	S	--	--	--
[51]	AX-OS	S	NS	NS	NS: LDL cholesterol ^#^, fasting insulin and glucose, stool consistency
[52]	GOS	--	--	--	S: Total cholesterol and insulin
					NS: LDL, and HDL cholesterol, triglycerides, or fasting glucose
[53]	AX-OS	S	--	NS	NS: GI symptoms
[54]	X-OS	NS	--	NS	NS: Fecal pH, GI symptoms
[55]	GOS	--	NS	S	S: Satiety, total energy intake
					NS: Weight/Adiposity
[56]	GOS	S	NS	NS	--

Abbreviations: AX-OS, arabinoxylan-oligosaccharides; FOS, fructooligosaccharides; GOS, galactooligosaccharides; NS, no statistically significant health benefit was observed; S, a significant effect was observed; SB-OS, soybean oligosaccharides; X-OS, xylo-oligosaccharides; ^#^ No significant effect of intervention, but the effect was in a direction opposite to providing a health benefit.

## Supplementary Materials

The following are available online at http://www.mdpi.com/2072-6643/9/2/125/s1, Table S1: Inclusion/Exclusion Criteria. Supplementary Methods. Figure S1: Acute studies: weighted scatter plot of other microbiota outcomes by fiber group presents weighted scatter. The acute studies exclusively examine the short-term fermentation response by measuring SCFA concentration and/or breath gas excretion, most frequently in cereal fibers. Figure S2: Longer duration studies: weighted scatter plot of other microbiota outcomes by fiber group. Studies of greater duration examine more outcomes in a larger array of fiber types. Notably, however, there are no longer duration (>4 weeks) studies on resistant starch.

## References

[1] Codex Alimentarius Commission (2008). Codex Alimentarius Commission Report of the 30th Session of the Codex Committee on Nutrition and Foods for Special Dietary Uses.

[2] Jones J.M. (2014). CODEX-aligned dietary fiber definitions help to bridge the “fiber gap”. Nutr. J..

[3] Dahl W.J., Stewart M.L. (2015). Position of the Academy of Nutrition and Dietetics: Health implications of dietary fiber. J. Acad. Nutr. Diet..

[4] Slavin J. (2013). Fiber and prebiotics: Mechanisms and health benefits. Nutrients.

[5] Conlon M.A., Bird A.R. (2014). The impact of diet and lifestyle on gut microbiota and human health. Nutrients.

[6] O’Hara A.M., Shanahan F. (2006). The gut flora as a forgotten organ. EMBO Rep..

[7] Nicholson J.K., Holmes E., Kinross J., Burcelin R., Gibson G., Jia W., Pettersson S. (2012). Host-gut microbiota metabolic interactions. Science.

[8] Hooper L.V., Littman D.R., Macpherson A.J. (2012). Interactions between the microbiota and the immune system. Science.

[9] Clemente J.C., Ursell L.K., Parfrey L.W., Knight R. (2012). The impact of the gut microbiota on human health: An integrative view. Cell.

[10] Flint H.J., Duncan S.H., Scott K.P., Louis P. (2015). Links between diet, gut microbiota composition and gut metabolism. Proc. Nutr. Soc..

[11] Blumberg R., Powrie F. (2012). Microbiota, disease, and back to health: A metastable journey. Sci. Transl. Med..

[12] Flint H.J. (2012). The impact of nutrition on the human microbiome. Nutr. Rev..

[13] David L.A., Maurice C.F., Carmody R.N., Gootenberg D.B., Button J.E., Wolfe B.E., Ling A.V., Devlin A.S., Varma Y., Fischbach M.A. (2014). Diet rapidly and reproducibly alters the human gut microbiome. Nature.

[14] Belenguer A., Duncan S.H., Calder A.G., Holtrop G., Louis P., Lobley G.E., Flint H.J. (2006). Two routes of metabolic cross-feeding between *Bifidobacterium adolescentis* and butyrate-producing anaerobes from the human gut. Appl. Environ. Microbiol..

[15] Falony G., Vlachou A., Verbrugghe K., De Vuyst L. (2006). Cross-feeding between *Bifidobacterium longum* BB536 and acetate-converting, butyrate-producing colon bacteria during growth on oligofructose. Appl. Environ. Microbiol..

[16] El Oufir L., Flourié B., Bruley des Varannes S., Barry J.L., Cloarec D., Bornet F., Galmiche J.P. (1996). Relations between transit time, fermentation products, and hydrogen consuming flora in healthy humans. Gut.

[17] Stephen A.M., Wiggins H.S., Cummings J.H. (1987). Effect of changing transit time on colonic microbial metabolism in man. Gut.

[18] Lewis S.J., Heaton K.W. (1997). Increasing butyrate concentration in the distal colon by accelerating intestinal transit. Gut.

[19] Graf D., Di Cagno R., Fåk F., Flint H.J., Nyman M., Saarela M., Watzl B. (2015). Contribution of diet to the composition of the human gut microbiota. Microb. Ecol. Health Dis..

[20] Den Besten G., van Eunen K., Groen A.K., Venema K., Reijngoud D.-J., Bakker B.M. (2013). The role of short-chain fatty acids in the interplay between diet, gut microbiota, and host energy metabolism. J. Lipid Res..

[21] Duncan S.H., Louis P., Thomson J.M., Flint H.J. (2009). The role of pH in determining the species composition of the human colonic microbiota. Environ. Microbiol..

[22] Samuel B.S., Shaito A., Motoike T., Rey F.E., Backhed F., Manchester J.K., Hammer R.E., Williams S.C., Crowley J., Yanagisawa M. (2008). Effects of the gut microbiota on host adiposity are modulated by the short-chain fatty-acid binding G protein-coupled receptor, Gpr41. Proc. Natl. Acad. Sci. USA.

[23] Wong J.M.W., de Souza R., Kendall C.W.C., Emam A., Jenkins D.J.A. (2006). Colonic health: Fermentation and short chain fatty acids. J. Clin. Gastroenterol..

[24] Tan J., McKenzie C., Potamitis M., Thorburn A.N., Mackay C.R., Macia L. (2014). The role of short-chain fatty acids in health and disease. Adv. Immunol..

[25] Birt D.F., Boylston T., Hendrich S., Jane J.-L., Hollis J., Li L., McClelland J., Moore S., Phillips G.J., Rowling M. (2013). Resistant starch: Promise for improving human health. Adv. Nutr. Int. Rev. J..

[26] Hamer H.M., Jonkers D., Venema K., Vanhoutvin S., Troost F.J., Brummer R.-J. (2008). Review article: The role of butyrate on colonic function. Aliment. Pharmacol. Ther..

[27] Flint H.J., Scott K.P., Louis P., Duncan S.H. (2012). The role of the gut microbiota in nutrition and health. Nat. Rev. Gastroenterol. Hepatol..

[28] Hamaker B.R., Tuncil Y.E. (2014). A perspective on the complexity of dietary fiber structures and their potential effect on the gut microbiota. J. Mol. Biol..

[29] Althuis M.D., Weed D.L. (2013). Evidence mapping: Methodologic foundations and application to intervention and observational research on sugar-sweetened beverages and health outcomes. Am. J. Clin. Nutr..

[30] Bragge P., Clavisi O., Turner T., Tavender E., Collie A., Gruen R.L. (2011). The global evidence mapping initiative: Scoping research in broad topic areas. BMC Med. Res. Methodol..

[31] Hetrick S.E., Parker A.G., Callahan P., Purcell R. (2010). Evidence mapping: Illustrating an emerging methodology to improve evidence-based practice in youth mental health. J. Eval. Clin. Pract..

[32] Wang D.D., Shams-White M., Bright O.J.M., Parrott J.S., Chung M. (2016). Creating a literature database of low-calorie sweeteners and health studies: Evidence mapping. BMC Med. Res. Methodol..

[33] Livingston K.A., Chung M., Sawicki C.M., Lyle B.J., Wang D.D., Roberts S.B., McKeown N.M. (2016). Development of a publicly available, comprehensive database of fiber and health outcomes: Rationale and methods. PLoS ONE.

[34] McKeown N.M., Chung M., Livingston K.A., Sawicki C.M., Wang D.D., Blakeley C., Jia Y., Baruch N., Karlsen M., Brown C. Project: Diet-Related Fibers and Human Health Outcomes, Version 1 (Retired Version). http://srdr.ahrq.gov/projects/564.

[35] Bouhnik Y., Flourié B., Riottot M., Bisetti N., Gailing M.F., Guibert A., Bornet F., Rambaud J.C. (1996). Effects of fructo-oligosaccharides ingestion on fecal bifidobacteria and selected metabolic indexes of colon carcinogenesis in healthy humans. Nutr. Cancer.

[36] Bouhnik Y., Vahedi K., Achour L., Attar A., Salfati J., Pochart P., Marteau P., Flourié B., Bornet F., Rambaud J.C. (1999). Short-chain fructo-oligosaccharide administration dose-dependently increases fecal bifidobacteria in healthy humans. J. Nutr..

[37] Tannock G.W., Munro K., Bibiloni R., Simon M.A., Hargreaves P., Gopal P., Harmsen H., Welling G. (2004). Impact of consumption of oligosaccharide-containing biscuits on the fecal microbiota of humans. Appl. Environ. Microbiol..

[38] Bouhnik Y., Raskine L., Simoneau G., Vicaut E., Neut C., Flourié B., Brouns F., Bornet F.R. (2004). The capacity of nondigestible carbohydrates to stimulate fecal bifidobacteria in healthy humans: A double-blind, randomized, placebo-controlled, parallel-group, dose-response relation study. Am. J. Clin. Nutr..

[39] Clavel T., Fallani M., Lepage P., Levenez F., Mathey J., Rochet V., Sérézat M., Sutren M., Henderson G., Bennetau-Pelissero C. (2005). Isoflavones and functional foods alter the dominant intestinal microbiota in postmenopausal women. J. Nutr..

[40] Ten Bruggencate S.J.M., Bovee-Oudenhoven I.M.J., Lettink-Wissink M.L.G., Katan M.B., van der Meer R. (2006). Dietary fructooligosaccharides affect intestinal barrier function in healthy men. J. Nutr..

[41] Bouhnik Y., Raskine L., Simoneau G., Paineau D., Bornet F. (2006). The capacity of short-chain fructo-oligosaccharides to stimulate faecal bifidobacteria: A dose-response relationship study in healthy humans. Nutr. J..

[42] Depeint F., Tzortzis G., Vulevic J., I’anson K., Gibson G.R. (2008). Prebiotic evaluation of a novel galactooligosaccharide mixture produced by the enzymatic activity of *Bifidobacterium bifidum* NCIMB 41171, in healthy humans: A randomized, double-blind, crossover, placebo-controlled intervention study. Am. J. Clin. Nutr..

[43] Vulevic J., Drakoularakou A., Yaqoob P., Tzortzis G., Gibson G.R. (2008). Modulation of the fecal microflora profile and immune function by a novel trans-galactooligosaccharide mixture (B-GOS) in healthy elderly volunteers. Am. J. Clin. Nutr..

[44] Mitsou E.K., Turunen K., Anapliotis P., Zisi D., Spiliotis V., Kyriacou A. (2009). Impact of a jelly containing short-chain fructo-oligosaccharides and *Sideritis euboea* extract on human faecal microbiota. Int. J. Food Microbiol..

[45] Cloetens L., Broekaert W.F., Delaedt Y., Ollevier F., Courtin C.M., Delcour J.A., Rutgeerts P., Verbeke K. (2010). Tolerance of arabinoxylan-oligosaccharides and their prebiotic activity in healthy subjects: A randomised, placebo-controlled cross-over study. Br. J. Nutr..

[46] Walton G.E., van den Heuvel E.G.H.M., Kosters M.H.W., Rastall R.A., Tuohy K.M., Gibson G.R. (2012). A randomised crossover study investigating the effects of galacto-oligosaccharides on the faecal microbiota in men and women over 50 years of age. Br. J. Nutr..

[47] Lecerf J.-M., Dépeint F., Clerc E., Dugenet Y., Niamba C.N., Rhazi L., Cayzeele A., Abdelnour G., Jaruga A., Younes H. (2012). Xylo-oligosaccharide (XOS) in combination with inulin modulates both the intestinal environment and immune status in healthy subjects, while XOS alone only shows prebiotic properties. Br. J. Nutr..

[48] Damen B., Cloetens L., Broekaert W.F., François I., Lescroart O., Trogh I., Arnaut F., Welling G.W., Wijffels J., Delcour J.A. (2012). Consumption of breads containing in situ-produced arabinoxylan oligosaccharides alters gastrointestinal effects in healthy volunteers. J. Nutr..

[49] François I.E.J.A., Lescroart O., Veraverbeke W.S., Marzorati M., Possemiers S., Evenepoel P., Hamer H., Houben E., Windey K., Welling G.W. (2012). Effects of a wheat bran extract containing arabinoxylan oligosaccharides on gastrointestinal health parameters in healthy adult human volunteers: A double-blind, randomised, placebo-controlled, cross-over trial. Br. J. Nutr..

[50] Walton G.E., Lu C., Trogh I., Arnaut F., Gibson G.R. (2012). A randomised, double-blind, placebo controlled cross-over study to determine the gastrointestinal effects of consumption of arabinoxylan-oligosaccharides enriched bread in healthy volunteers. Nutr. J..

[51] Maki K.C., Gibson G.R., Dickmann R.S., Kendall C.W.C., Chen C.-Y.O., Costabile A., Comelli E.M., McKay D.L., Almeida N.G., Jenkins D. (2012). Digestive and physiologic effects of a wheat bran extract, arabino-xylan-oligosaccharide, in breakfast cereal. Nutrition.

[52] Vulevic J., Juric A., Tzortzis G., Gibson G.R. (2013). A mixture of trans-galactooligosaccharides reduces markers of metabolic syndrome and modulates the fecal microbiota and immune function of overweight adults. J. Nutr..

[53] François I.E.J.A., Lescroart O., Veraverbeke W.S., Marzorati M., Possemiers S., Hamer H., Windey K., Welling G.W., Delcour J.A., Courtin C.M. (2014). Effects of wheat bran extract containing arabinoxylan oligosaccharides on gastrointestinal parameters in healthy preadolescent children. J. Pediatr. Gastroenterol. Nutr..

[54] Finegold S.M., Li Z., Summanen P.H., Downes J., Thames G., Corbett K., Dowd S., Krak M., Heber D. (2014). Xylooligosaccharide increases bifidobacteria but not lactobacilli in human gut microbiota. Food Funct..

[55] Morel F.B., Dai Q., Ni J., Thomas D., Parnet P., Fança-Berthon P. (2015). α-Galacto-oligosaccharides dose-dependently reduce appetite and decrease inflammation in overweight adults. J. Nutr..

[56] Vulevic J., Juric A., Walton G.E., Claus S.P., Tzortzis G., Toward R.E., Gibson G.R. (2015). Influence of galacto-oligosaccharide mixture (B-GOS) on gut microbiota, immune parameters and metabonomics in elderly persons. Br. J. Nutr..

[57] Hur I.Y., Reicks M. (2012). Relationship between whole-grain intake, chronic disease risk indicators, and weight status among adolescents in the National Health and Nutrition Examination Survey, 1999–2004. J. Acad. Nutr. Diet..

[58] Ley R.E., Bäckhed F., Turnbaugh P., Lozupone C.A., Knight R.D., Gordon J.I. (2005). Obesity alters gut microbial ecology. Proc. Natl. Acad. Sci. USA.

[59] Kalliomäki M., Collado M.C., Salminen S., Isolauri E. (2008). Early differences in fecal microbiota composition in children may predict overweight. Am. J. Clin. Nutr..

[60] Carbohydrates in Human Nutrition (1997). Report of a Joint FAO/WHO Expert Consultation.

[61] American Association of Cereal Chemists (AACC) (2001). The Definition of Dietary Fiber. Report of the Dietary Fiber Definition Committee to the Board of Directors of the American Association of Cereal Chemists. Cereal Foods World.

[62] Valcheva R., Dieleman L.A. (2016). Prebiotics: Definition and protective mechanisms. Best Pract. Res. Clin. Gastroenterol..

[63] Bindels L.B., Delzenne N.M., Cani P.D., Walter J. (2015). Towards a more comprehensive concept for prebiotics. Nat. Rev. Gastroenterol. Hepatol..

[64] Cummings J.H., Antoine J.-M., Azpiroz F., Bourdet-Sicard R., Brandtzaeg P., Calder P.C., Gibson G.R., Guarner F., Isolauri E., Pannemans D. (2004). PASSCLAIM—Gut health and immunity. Eur. J. Nutr..

[65] Meyer D., Cho S.S., Almeida N. (2012). Inunlin, gut microbes, and health. Dietary Fiber and Health.

[66] Tojo R., Suárez A., Clemente M.G., de los Reyes-Gavilán C.G., Margolles A., Gueimonde M., Ruas-Madiedo P. (2014). Intestinal microbiota in health and disease: Role of bifidobacteria in gut homeostasis. World J. Gastroenterol..

[67] McRorie J.W. (2015). Evidence-based approach to fiber supplements and clinically meaningful health benefits, part 1: What to look for and how to recommend an effective fiber therapy. Nutr. Today.

[68] McRorie J.W. (2015). Evidence-based approach to fiber supplements and clinically meaningful health benefits, part 2: What to look for and how to recommend an effective fiber therapy. Nutr. Today.

[69] Mcrorie J.W., Fahey G.C. (2013). A review of gastrointestinal physiology and the mechanisms underlying the health benefits of dietary fiber: Matching an effective fiber with specific patient needs. Clin. Nurs. Stud..

[70] McRorie J.W., McKeown N.M. (2017). Understanding the physics of functional fibers in the gastrointestinal tract: An evidence-based approach to resolving enduring misconceptions about insoluble and soluble fiber. J. Acad. Nutr. Diet..

[71] 2015–2020 Dietary Guidelines—Health.gov. https://health.gov/dietaryguidelines/2015/guidelines/.

[72] Okarter N., Liu R.H. (2010). Health benefits of whole grain phytochemicals. Crit. Rev. Food Sci. Nutr..

[73] Vitaglione P., Mennella I., Ferracane R., Rivellese A.A., Giacco R., Ercolini D., Gibbons S.M., La Storia A., Gilbert J.A., Jonnalagadda S. (2015). Whole-grain wheat consumption reduces inflammation in a randomized controlled trial on overweight and obese subjects with unhealthy dietary and lifestyle behaviors: Role of polyphenols bound to cereal dietary fiber. Am. J. Clin. Nutr..

