# Supplementary material for: Dietary Fiber and the Human Gut Microbiota: Application of Evidence Mapping Methodology
Source: Nutrients. 2017 Feb 10;9(2):125. doi: 10.3390/nu9020125 (PMC5331556; doi:10.3390/nu9020125)
Supplement: Supplementary file 1 [file nutrients-09-00125-s001.docx]

Supplementary Materials: Dietary Fiber and the Human Gut Microbiota: Application of Evidence Mapping Methodology

Caleigh M. Sawicki, Kara A. Livingston, Martin Obin, Susan B. Roberts, Mei Chung
and Nicola M. McKeown

**Table S1.** Inclusion/Exclusion Criteria.

| **Inclusion Criteria** | **Exclusion Criteria** |
| --- | --- |
| - Published 1946–May 2016 - Published in English - Abstract contained a fiber term and one of the following Vahouny health outcomes:   1. Total & LDL cholesterol  2. Post-prandial glucose & insulin  3. Blood pressure  4. Fecal bulk & laxation  5. Transit time  6. Colonic fermentation & SCFA concentration  7. Modulation of colonic microflora  8. Weight/Adiposity  9. Increased satiety | - Reviews, bibliographies, case reports - Observational studies - Intervention was via tube feeding or enteral nutrition - Population was infants (<3 years), pregnant or breastfeeding women, has any type of cancer, bowel disease, renal failure, or other chronic disease condition - Intervention has no concurrent control - Fiber dose not clearly reported - Intervention not sufficiently controlled to isolate the effect of fiber - Animal-only studies - In vitro studies |

Supplemental Methods. Keywords and MeSH headings used to identify microbiota outcomes in the development of the fiber database

1. Colonic fermentation and SCFA concentration

Fermentation: Fermentations

Fatty Acids, Volatile: Volatile Fatty Acids; Fatty Acids, Short-Chain; Fatty Acids, Short Chain; Short-Chain Fatty Acids

Acetates: Acetic Acid Esters; Esters, Acetic Acid; Acetic Acids; Acids, Acetic

Butyrates: n-Butyrate; n Butyrate; Butyrate; Butyric Acids; Acids, Butyric; Butanoic Acids; Acids, Butanoic

Caproates: Hexanoates; Hexanoic Acids; Acids, Hexanoic; Caproic Acid; Acid, Caproic; Capronic Acid; Acid, Capronic

Propionates: Propanoates; Propionic Acids; Acids, Propionic; Propanoic Acids; Acids, Propanoic

Valerates: Pentanoates

2. Modulation of colonic microflora

Metagenome: Metagenomes; Microbiome; Microbiomes; Microbiota; Microbiotas; Human Microbiome; Human Microbiomes; Microbiomes, Human; Microbiome, Human

(Not a Mesh term): [Colonic, colon, intestinal, gastrointestinal, bowel, gut] microflora; microbiota; bacteria; microorganisms; microbial [population, communities, composition]

(Not a Mesh term): autochthonous flora; indigenous flora; allochthonous flora; transient flora

(Not a Mesh term): [Fecal, stool] pH

Bifidobacteria

Lactobacillus/lactobacilli: helveticus, casei, leichmannii, reuteri, brevis, plantarum, delbrueckii, acidophilus, rhamnosus, fermentum


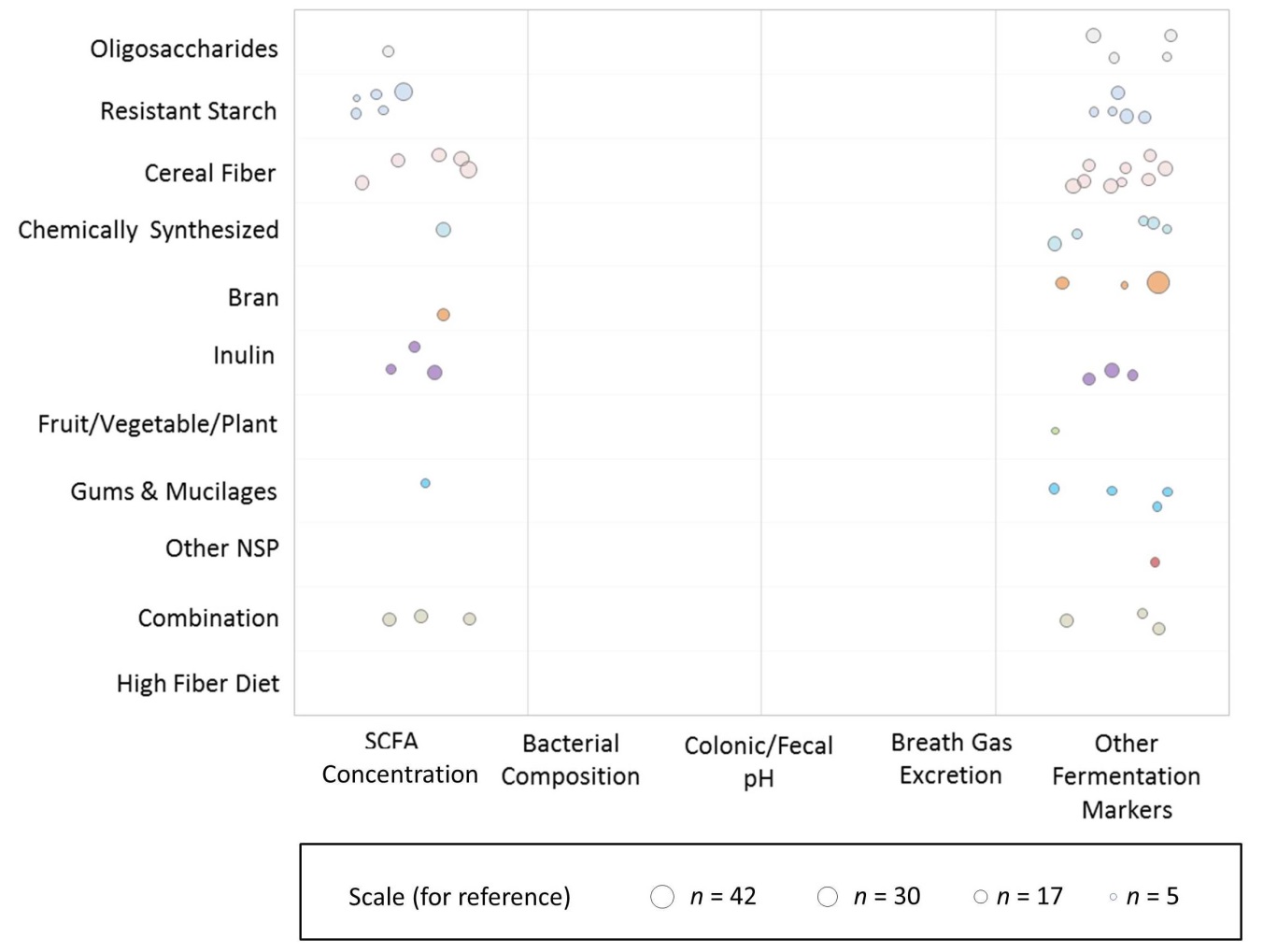


**Figure S1.** Acute studies**:** weighted scatter plot of other microbiota outcomes by fiber group. Each bubble in the plot represents a single publication with the size of the bubble corresponding to the study sample size. Studies may be represented more than once throughout the plot if multiple fiber interventions or outcomes were reported, but are not repeated within any single cross-sectional area. Note that the outcome effect is not represented in this graphic i.e., this does not reflect the effect of the fiber on the outcome.


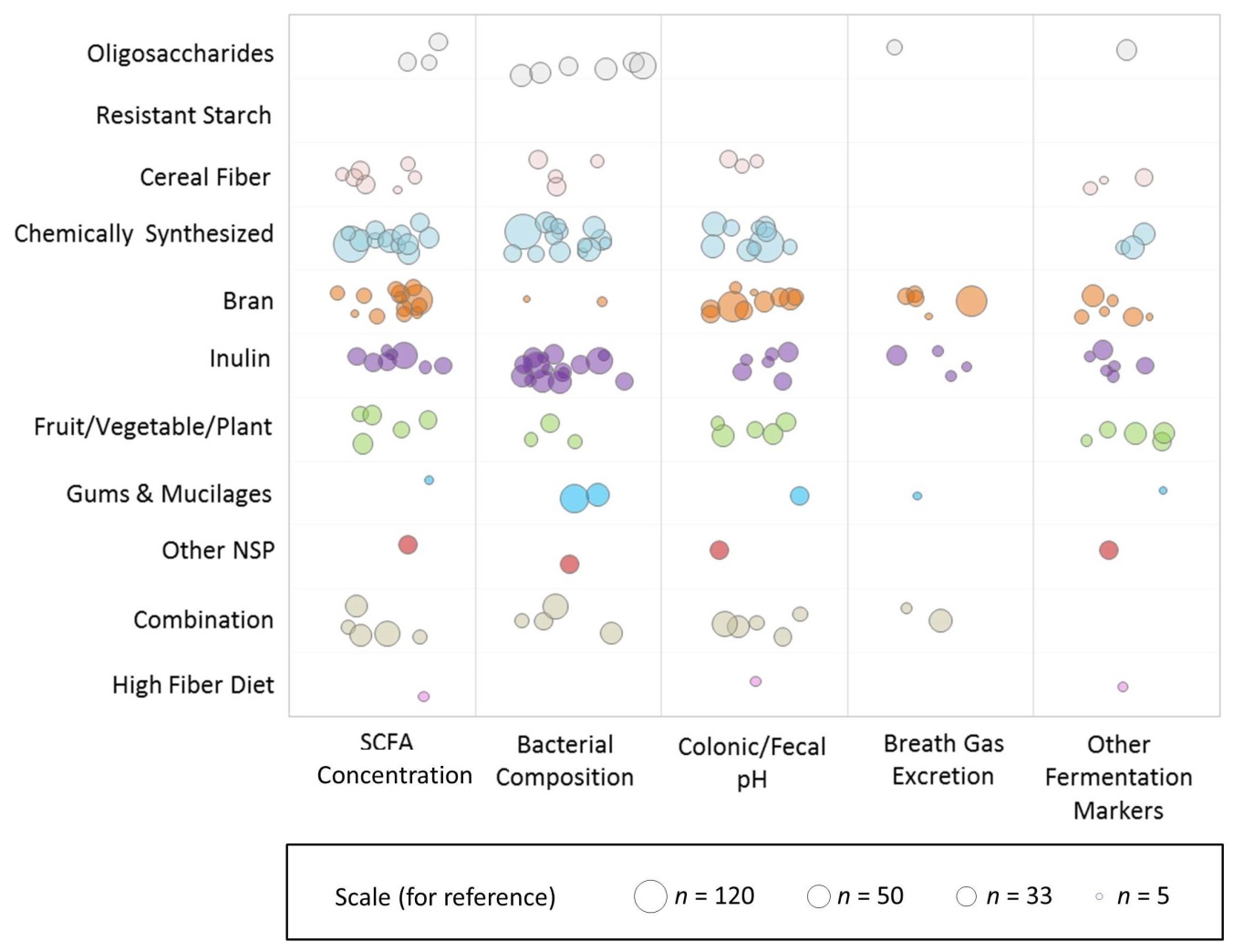


**Figure S2.** Longer duration studies**:** weighted scatter plot of other microbiota outcomes by fiber group. Each bubble in the plot represents a single publication with the size of the bubble corresponding to the study sample size. Studies may be represented more than once throughout the plot if multiple fiber interventions or outcomes were reported, but are not repeated within any single cross-sectional area. Note that the outcome effect is not represented in this graphic i.e., this does not reflect the effect of the fiber on the outcome.
